# Revisiting the radiation of *Gazella arabica* on the Arabian Peninsula and testing the suitability of captive breeding stock for reintroduction, using mitochondrial and nuclear markers

**DOI:** 10.1016/j.sjbs.2023.103823

**Published:** 2023-10-05

**Authors:** Mohamed Al Mutairi, Hannes Lerp, Naif Al Hanosh, William Macasero, Mohammed F. Al Beshr, Torsten Wronski

**Affiliations:** a Terrestrial Wildlife Conservation Department, National Centre for Wildlife (NCW), P.O. Box 61681, Riyadh 11575, Saudi Arabia; b Natural History Collections, Museum Wiesbaden, Friedrich-Ebert-Allee 2, 65185 Wiesbaden, Germany; c King Khalid Wildlife Research Centre, National Centre for Wildlife (NCW), P.O. Box 61681, Riyadh 11575, Saudi Arabia; d King Saud University, Faculty of Science, Department of Zoology, P.O Box 800, Riyadh 11421, Saudi Arabia; e Faculty of Science, School of Biological and Environmental Sciences, Liverpool John Moores University, Liverpool L3 3AF, UK

**Keywords:** Arabian gazelle, Radiation, Restricted gene flow, Ex-situ breeding, Reintroduction

## Abstract

Today, the Arabian gazelle (*G. arabica*) occurs only in small, scattered populations on the Arabian Peninsula and is classified as ‘vulnerable’, due to intensive hunting and competition with livestock. The taxonomy of this threatened species is still under debate, hampering conservation efforts while ex-situ breeding programs could be an appropriate conservation measure to prevent the species from going extinct. In our study, we attempted to elucidate the radiation of *G. arabica* on the Arabian Peninsula, and to ask whether the population genetic structure allows to distinguish between discrete conservation units. We used mitochondrial markers, microsatellite markers, and three intron markers to identify conservation units, to match them with genotypes found in the captive breeding stock held in Saudi Arabia, and to ensure that genotype diversity of potential founder individuals corresponds to that prevailing at targeted reintroduction sites. The sequence divergence was low among nuclear and mitochondrial markers, with gazelles originating from the north of the Arabian Peninsula showing the largest diversity, while south-western and eastern populations showed a decreased diversity. A haplotype network based on the relatively heterogeneous cytochrome *b* gene found no signs of a prolonged separate evolutionary history of any investigated mainland population, suggesting limitations of gene-flow after the colonization of the Arabian Peninsula leading to a founder effect-like distribution of mitochondrial haplotypes. The ex-situ breeding population held in Saudi Arabia showed a good haplotype diversity, underlining its general suitability for reintroductions. However, it is recommended that genetic data of founders should be assessed prior to future reintroduction.

## 1 Introduction

**Fig. 1:**
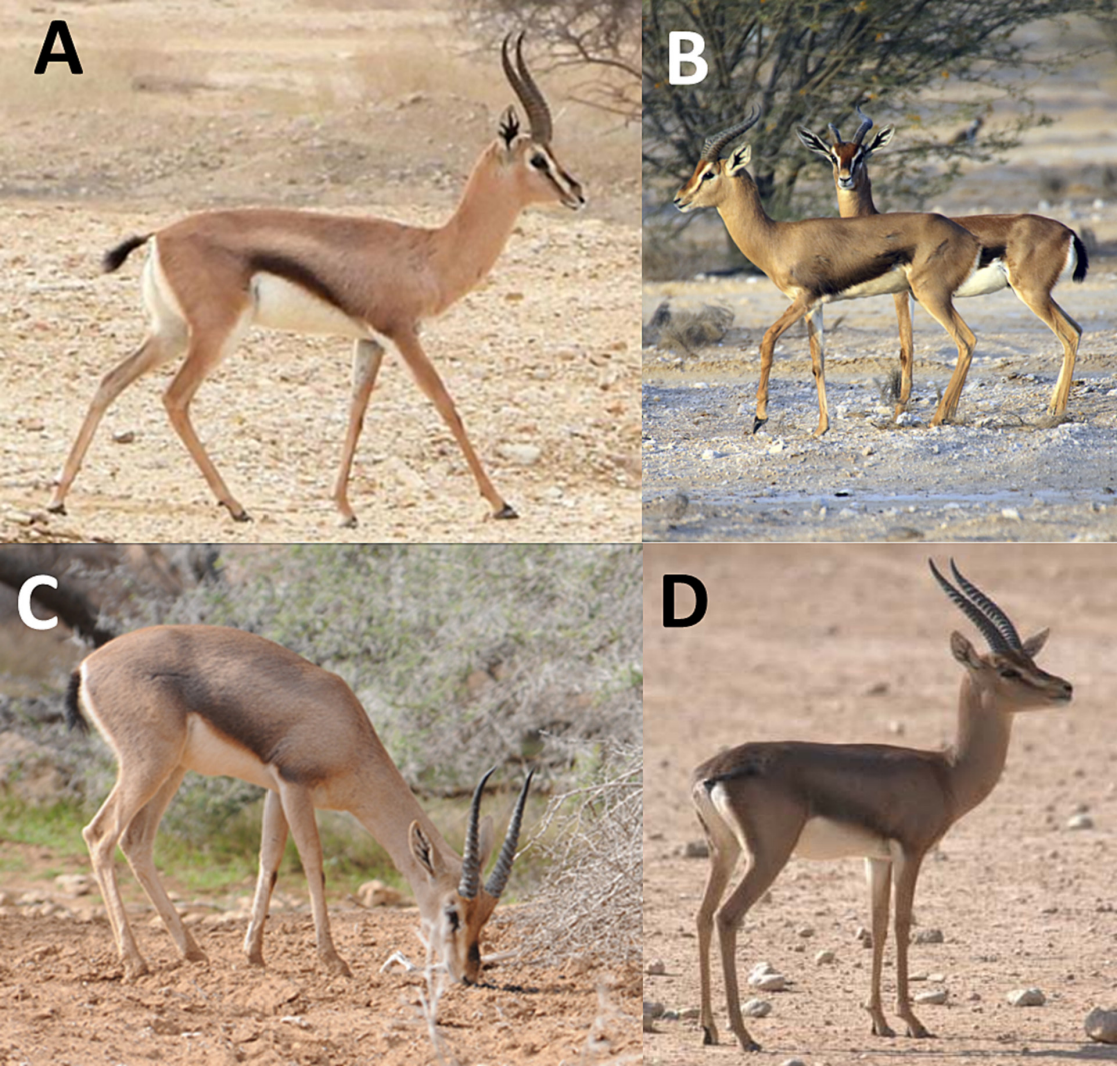
Adult male Arabian gazelles (*Gazella arabica*) from (A) the Arava Valley in southern Israel (Photo B. Shalmon), (B) the Farasan Islands, south-western Saudi Arabia (Photo T. Wronski), (C) Al Ain Nature Reserve, UAE (Photo O. Fragman-Sapir), and (D) the captive breeding stock at King Khalid Wildlife Research Center in Saudi Arabia (Photo T. Butynski).

Genetic and paleontological evidence indicates that ‘true or slender gazelles’ (genus *Gazella*) first emerged 14–6.3 Ma ago, in the late Miocene of Africa (Vrba, 1985, Hassanin et al., 2012, Lerp et al., 2013a). One of the latest speciation events within the genus was the separation of Arabian (*Gazella arabica,* Lichtenstein 1827) and mountain gazelles (*G. gazella,* Pallas 1776) during the mid-Pleistocene of the Middle East (0.9–1.7 Ma ago; Lerp et al., 2013a, b). During that period, the Middle East was pivotal to adaptive radiations triggered by biogeographical heterogeneity and a prominent biotic exchange between African, Asian, and European faunal elements (Tchernov, 1988). Taxa immigrating from Africa, such as the true gazelles, experienced a vast speciation process, enabling them to invade cooler and more humid areas, such as the Mediterranean shrublands (maquis) of the Levant (*G. gazella*) or the arid and rocky habitats of the Arabian Peninsula (*G. arabica*; Lerp et al., 2013b). Today, Arabian gazelles occur in scattered populations across the Arabian Peninsula reaching from the Arava Valley in southern Israel through western Saudi Arabia, Yemen, Oman, into the United Arab Emirates (Mallon and Kingswood, 2001, IUCN SSC Antelope Specialist Group, 2017). Morphological analysis of *G. arabica* suggested several subspecies (Groves and Grubb, 2011), but the identity, taxonomy, and nomenclature of these taxa is still a matter of debate. Recent studies (Hadas et al., 2015, Lerp et al., 2014, Fadakar et al., 2021) elucidated the phylogenetic status of the Acacia gazelle (*G. arabica acacia*e; Mendelssohn et al., 1997; Fig. 1A), and the island populations of Farasan gazelle (*G. arabica farasani*; Thouless and Bassri, 1991; Fig. 1B) and Farur gazelle (*G. arabica dareshourii*; Karami and Groves, 1993), whereby only the later was confirmed an evolutionary significant unit (*sensu* Ryder, 1986, Ryder, 1987). Other taxa were suspected to be domesticated forms of *G. arabica* (e.g., *G. muscatensis* or *G. erlangeri*; Wronski et al., 2017), or were synonymized with *G. arabica* (e.g., *Antilope cora*; Bärmann et al., 2013a, Bärmann et al., 2013b, Bärmann et al., 2014; Fig. 1C). However, phylogenetic analysis suggested consistent differences between Farasan Island and Arabian mainland populations (Lerp et al., 2014), but also between the eastern and western mainland populations, insinuating that two conservation units (*sensu* Ryder, 1986, Ryder, 1987) may exist (Bärmann et al., 2013a). In this study, we first attempted to further elucidate the radiation of *G. arabica* on the Arabian Peninsula and the Farasan Islands, asking whether the population genetic structure indicates a speciation within *G. arabica*, and whether evolutionary significant units—or better distinct management or conservation units—can be identified.

The total wild population of Arabian gazelles is currently estimated to be less than 7,000 individuals, resulting in an IUCN Red List classification as ‘vulnerable’ (IUCN/SSC Antelope Specialist Group, 2017). In Saudi Arabia, *G. arabica* still occurs in good numbers on the Farasan Islands (Soares and Wronski, 2021) and in some small, scattered populations in the north and west of the country (Al Hazmi and Ghandour, 1992, Seddon et al., 1997, Boug et al., 2012, Wronski and Butynski, 2014). Apart from these original populations, Arabian gazelles were introduced to the Uruq Bani Ma'arid (Islam et al., 2011) and Mahazat as-Sayd Protected Areas (Sher Shah et al., 2013, Islam et al., 2014) as well as to the Ibex Reserve (Dunham et al., 1993). At present, significant captive populations of *G. arabica* are kept at Al Ain Wildlife Park in the UAE, Al Wabra Wildlife Preservation (AWWP) in Qatar and at King Khalid Wildlife Research Centre (KKWRC) in Saudi Arabia (Blacket, 2001, Blacket et al., 2001, Hammer, 2010, Chege et al., 2014). The KKWRC breeding stock is composed of a mix of gazelles descending from animals of geographically distant populations across Saudi Arabia (Hammond et al. 2001, this study; Fig. 1D) and mainly serves conservation purposes, i.e., the release of gazelles into the protected areas of Saudi Arabia (Wacher and Kichenside, 1998, Mohammed, 2002). Given that evolutionary significant units within the *G. arabica* population may exist, it is imperative for future reintroductions to choose the right individuals from the captive breeding stock for release into the wild. Founders should show traits that are based on morphology, physiology, and genetic origin, and that should be assessed as appropriate through comparison with the original wild populations (IUCN/SSC, 2013). The second objective of our present study is, therefore, to match previously identified management or conservation units with the genotypes found in the KKWRC breeding stock and to ensure that genotype diversity of potential founder individuals from KKWRC corresponds to that prevailing in the targeted release region.

## 2 Materials and methods

### 2.1 Data collection

We analysed nuclear and mitochondrial sequence data from 91 specimens of wild and captive *G. arabica* and five specimens of two outgroup species, i.e., *G. gazella* and *G. dorcas*. In total, we used 225 sequences of which 50 were previously available in Genbank and 175 that were additionally sequenced for this study (for Genbank accession numbers and sample information see Table S1 in ESM).

**Fig. 2:**
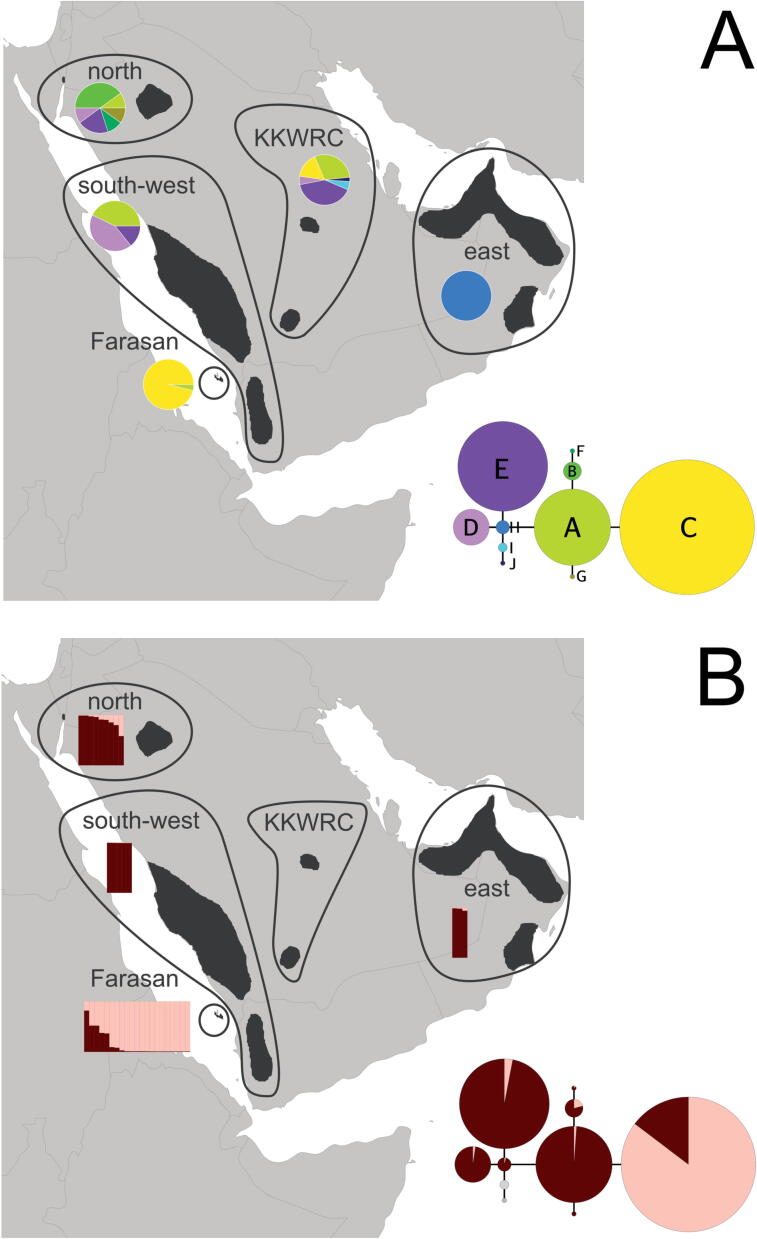
Distribution of haplotypes and population genetic clusters in the sampled individuals of *G. arabica*. (A) Relative frequency of different haplotypes within the geographic groups ‘north’, ‘south-west’, ‘east’, ‘Farasan’ and ‘unknown’. The latter corresponds to individuals held at King Khalid Wildlife Research Center (KKWRC) in the Kingdom of Saudi Arabia. The inserted haplotype network is based on the alignment of the mitochondrial cytochrome *b* gene. The sizes of the circles are proportional to the number of individuals sampled (with the circle of haplotype F representing one individual, while the circle of haplotype C representing 31 individuals). The segment size of the connecting lines is proportionate to the amount of mutations between the haplotypes (in most cases one). (B) Population genetic assignment inferred from microsatellite data presented by Lerp et al. (2014) for K = 2 clusters (two shades of red) and the samples used in this study. Each bar in the map represents one individual and is assigned to the area of origin. The inserted haplotype network shows the mean assignment score (Q) to each of the four clusters.

Newly collected samples were obtained from hairs and faeces, from dried skins or from blood samples taken during veterinary screening procedures and were sequenced at the laboratory of King Khalid Wildlife Research Centre (KKWRC) in Saudi Arabia. Most samples (N = 37) were obtained from captive individuals kept at KKWRC. Other samples were collected from wild animals in three geographic regions in which *G. arabica* naturally occurs (Table S1 in ESM, Fig. 2), i.e., the northern (N = 2), the south-western (N = 3) and the eastern region of the Arabian Peninsula (N = 1) as well as from the Farasan Islands (N = 25). Two samples were assembled from confiscated gazelles at the Akhoba Market in Jizan (south-western region).

DNA extraction was performed using the Qiagen DNeasy Blood & Tissue Kit and the Qiagen QIAamp DNA Stool Mini Kit following the instructions provided by the manufacturer. For the Polymerase chain reactions the primers KKWRC5 and KKWRC9 (Hundertmark, 2005) for the mitochondrial cytochrome *b* gene, and primers published in Lerp et al. (2016b) for the nuclear introns NLRP2, PANK4 and Smoc1 were used. PCR was performed in a 25-μl reaction volume using 0.1 µl Taq polymerase solution, 2.5 µl 10x PCR buffer, 2 µl 1 mM dNTPs, 0.75 µl 1.5 mM MgCl2 and 0.15 µl each for 25 μM of each primer. Cycle conditions were as follows: initial denaturation (180 s at 95 °C), followed by 35 cycle steps of 60 s at 94 °C, 60 s at 50 °C and 90 s at 72 °C, and lastly, a final extension step (600 s at 72 °C). We performed a gel electrophoresis to check whether the PCR was successful. Amplified PCR products were then Sanger sequenced by Macrogen, Inc. South Korea using the PCR primers.

### 2.2 Data analyses

To analyse molecular data, we created different subsets of sequences and data arrangements, depending on the kind of analysis performed:

(1) Phylogeographic analyses were performed using a cytochrome *b* alignment with a length of 391 base pairs of N = 88 *G. arabica* specimens, incorporating most available samples, especially those with known provenance, as well as N = 5 outgroup sequences (Table S1 in ESM). We constructed a statistical parsimony (SP) network using TCS v1.21 (Clement et al., 2000) with a connection limit of 95% such that no outgroup specimen would be connected. To compare the sequence divergence of cytochrome *b* and to identify possible speciation events between geographic groups of *G. arabica* (i.e., north, south-west, east and Farasan), we calculated pairwise Kimura 2-Parameter *p*-distances (K2P) using MEGA 11 (Tamura et al., 2021). K2P-values (x100) based on cytochrome *b* sequence data are known to give a good resolution in separating species if values are smaller than 1.5, while values larger than 2.5 indicate two distinct species (Tobe et al., 2010).
(2) For N = 42 specimens, population genetic data, inferred from a STRUCTURE analysis based on microsatellite length-polymorphism sequences, were available (for details see Lerp et al. (2014) and accompanying ESM). ΔK was highest for K = 2 predefined clusters (see Fig. 3 in Lerp et al. 2014), so we combined probabilistic assignments (Q-values) for K = 2 for each individual with haplotype groups inferred from analysis of cytochrome *b* to further evaluate possible differentiation between groups.

**Fig. 3:**
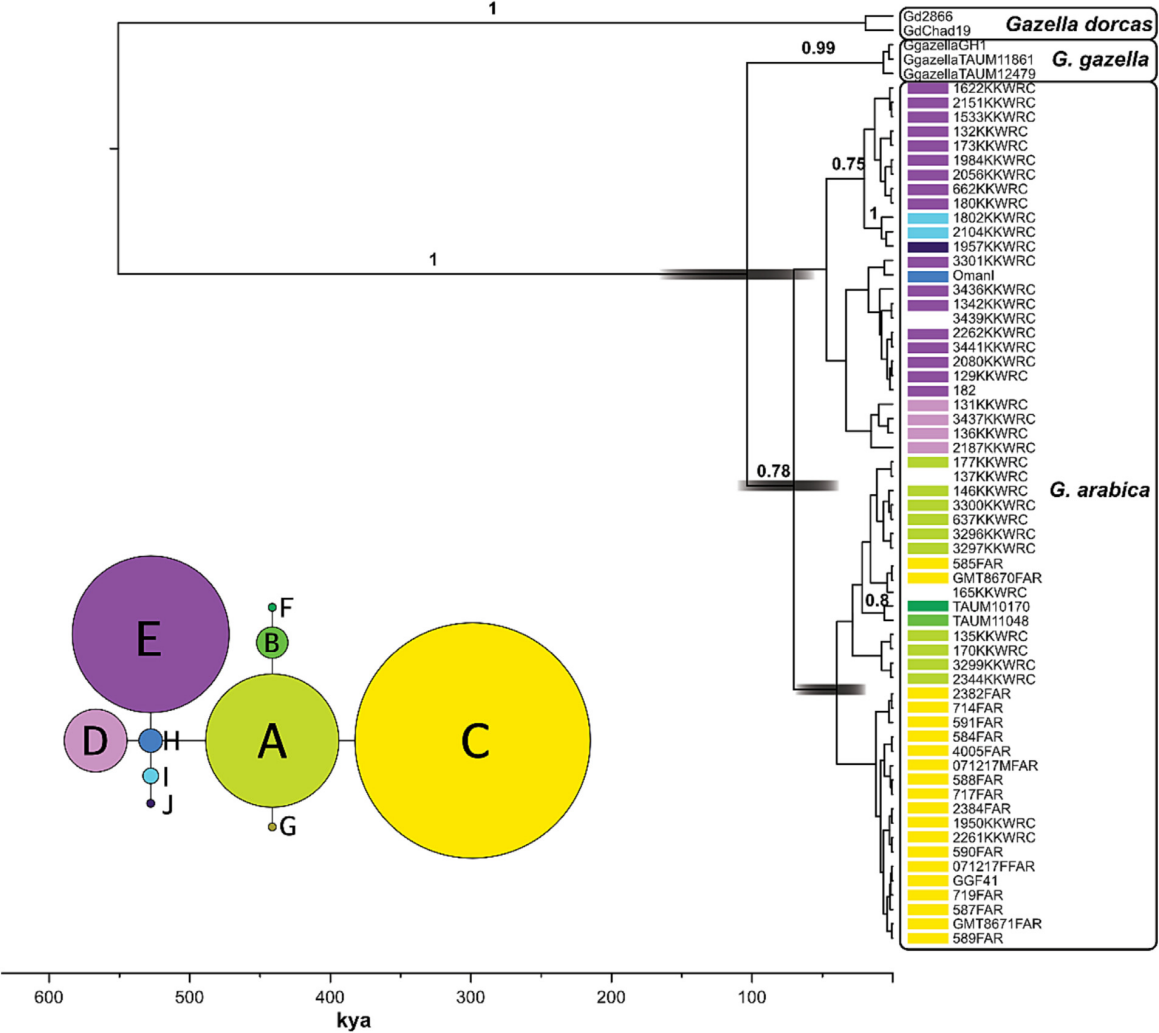
Phylogenetic tree of 65 samples of Gazella arabica and related taxa based on a concatenated alignment consisting of 2,032 nucleotides of three nuclear intron markers and mitochondrial Cytb. The tree was inferred with 5 × 10^7^ generations (10% burn-in) using the software BEAST 1.10.4 (Suchard et al., 2018). Above branches, posterior probability values ≥ 0.75 are reported. Node bars show the 95% credibility intervals of the divergence times of the respective phylogenetic split. Colour-coding of the G. arabica specimens correspond to the respective mitochondrial haplotypes inferred from statistical parsimony network analysis (inserted figure). No colour represents samples not included into the haplotype network.
(3) A phylogenetic analysis was conducted using a concatenated alignment (N = 65) of the mitochondrial sequence marker cytochrome *b* and the nuclear intron markers NLRP2, PANK4 and Smoc1 with a total length of 2032 base pairs. To make use of the best-fitting partitioning scheme and the respective nucleotide substitution models, we used the greedy search algorithm with PhyML (Guindon et al., 2010) implemented in PartitionFinder v.2.1.1 (Lanfear et al., 2012, Lanfear et al., 2017). We tested between different partitioning formats containing the cytochrome *b* protein-coding gene and three nuclear intron partitions. Models were selected by the AIC value. The optimal partitioning format included three partitions (with the respective models in brackets), i.e., cytochrome *b* (HKY), NLRP2 and Smoc1 (HKY + I), and PANK4 (GTR + I + G). This partition format was set in a Bayesian analysis performed in BEAST 1.10.4 (Suchard et al., 2018). No outgroup was defined beforehand. Furthermore, we used molecular clock data estimations inferred for the genus *Gazella* (Lerp et al., 2016b) and ran MC^3^ simulations with 5 × 10^7^ generations, discarding the first 10% of the runs as burn-in.

## 3 Results

To elucidate the geographic structure of our data, we created a haplotype network and plotted it onto a map of the Arabian Peninsula (Fig. 2A). Within our sampling, we were able to detect 10 haplotypes (A-J). The largest haplotype diversity was detected (i) among samples from the north of the distribution area, i.e., six haplotypes of which three were private and, (ii) within the captive breeding stock of KKWRC with six haplotypes of which two were private. In the south-western region, three haplotypes were present, all of which were also found in the northern region, as well as one haplotype (A) that was also found on the Farasan Islands. In most samples originating from the Farasan Islands (N = 25 of 26), we found a private haplotype (C) that was otherwise only detected in the captive breeding stock of KKWRC. This haplotype had only a single mutational step to haplotype A (Fig. 2A). In the east of the species’ distribution area, only one private haplotype (H) was detected. In the corresponding haplotype network (Fig. 2A) this haplotype lies in the centre of the network with only one mutational step to haplotypes A, D, E, and I. To evaluate potential speciation processes, we calculated the Kimura-2-parameter distances (x100; Table 1) and found species level divergence in *G. dorcas* compared to *G. gazella* and *G. arabica* (K2P > 4.7). The K2P-values of comparisons between *G. gazella* and geographic groups of *G. arabica* were larger than two indicating species-level divergence between both taxa. However, within the geographic groups of *G. arabica*, the K2P-values were all smaller than 1.5. Even if only individuals from the Farasan Islands with the unique haplotype C were considered, K2P-values increased only slightly.

**Table 1:** Kimura-2-parameter distances (x100) between different gazelle species (*G. dorcas* and *G. gazella*) and four populations (*G. arabica*). Values below the line include all samples for each population, while values above the line are distances of mainland populations and only those individuals from the Farasan Islands that have the unique Farasan haplotype C (Fig. 2A).

Looking at the available population genetic data (i.e., the probabilistic assignments (Q-values) published by Lerp et al. 2014), we found one dominant genotype that was present in all mainland haplotypes (Fig. 2B dark red cluster). A unique genotype was found in Farasan Island gazelles, which was almost entirely represented by the Farasan haplotype C (Fig. 2B pink cluster). However, the population genetic admixture of mainland DNA was also found in this haplotype, suggesting an ongoing introgression of genes into individuals carrying the ‘ancient’ Farasan haplotype.

The phylogenetic tree (Fig. 3), based on the concatenated alignment of cytochrome *b* and the intron markers NLRP2, PANK4 and Smoc1, indicated high posterior probability support for the clades comprising *G. dorcas* (PP = 1) and *G. gazella* (PP = 0.99). For the monophyly of *G. arabica,* a posterior probability of 0.78 was estimated with a divergence time from *G. gazella* during the Calabrian period (median: 100 kya 95%; HPD: 1666–55 kya). Within *G. arabica* one clade comprising the majority of Farasan samples (16 out of 19) and two captive specimens from KKWRC was uncovered with a divergence time of 39 kya (95%; HPP: 70–19 kya). However, this clade received only weak statistical support (PP = 0.64).

## 4 Discussion

### 4.1 General remarks

The genetic sampling of *G. arabica* specimens included in our study was rather homogenous with relatively low variation across the Arabian Peninsula. However, nuclear intron markers were not only proven to be capable of distinguishing between gazelle species (Lerp et al., 2016a) but also between entities within species, e.g., *G. dorcas* (Lerp et al., 2016a) and *G. arabica dareshurii* (Fadakar et al., 2021). Most of the genetic structure observed in our study was due to differences between cytochrome *b* sequences. This is because only females inherit mitochondrial genes, while the dispersal is usually male-biased (Dunham, 2000). Nowadays, gene flow between *G. arabica* populations is restricted, owing to large distances between scattered, isolated areas in which gazelles persist—usually protected areas or remote regions with no, or limited access for motor vehicles. In the past, however, it can be assumed that the distribution of Arabian gazelles on the Arabian Peninsula was continuous—at least along the mountainous areas of the West, South and East, enabling a constant gene flow over large distances or, at least, a link-up of populations through dispersing males. Otherwise, we would have unravelled signals of independent evolution in our data, i.e., visible mutations in the intron regions, more variable, heterogenous cytochrome *b* gene sequences and a population genetic separation between the sampling regions. Moreover, archaeological findings of excavated desert kites, used in pre-historic times to hunt migratory species, such as *Gazella marica* (Legge and Rowley-Conwy, 1987, Bar-Oz et al., 2011), suggest significantly higher population numbers and, therefore, generally higher rates of geneflow during those periods – not only among migrating populations such as the Arabian sand gazelle (*Gazella marica*) but also between more sedentary species like *Gazella dorcas* or *G. arabica*. According to Lerp et al. (2011), the implementation of desert kites corresponds to a sharp decline in population size about 10,000 years before present and marks the beginning of population fragmentations and thus the end of unhampered gene flow between populations.

### 4.2 Radiation of *G. arabica* on the Arabian Peninsula and the Farasan Islands

The largest variety of haplotypes in our study was found in the north of the Arabian Peninsula, suggesting that the place of origin of *G. arabica* was located in the East-Mediterranean Levant. This result corresponds to findings reported by Lerp et al. (2013a), who further proposed a subsequent southward dispersal along the western mountains of the Arabian Peninsula (Hejaz and Asir Mountains) during the cooler and wetter periods of the early Pleistocene. Consequently, Arabian gazelles from the south-west of the Peninsula showed a reduced haplotype diversity (N = 3) compared to that found in the north (Fig. 2A). Our data further suggest that with progressing colonization, Arabian gazelles eventually reached the eastern mountains of the Arabian Peninsula, i.e., the Hajar Mountains of Oman and the UAE. However, this likely has been a limited number of individuals that either during dispersal, or after reaching eastern Arabia, experienced a bottleneck, resulting into the unique haplotype H found in our study (Fig. 2A). However, classifying samples from the eastern part of Arabia as an evolutionary significant unit seems not to be justified for three reasons: (1) the unique haplotype H is situated in the centre of our haplotype network and, the respective K2P-values of are low (especially between south-west and east group). (2) The population genetic assignment based on data by Lerp et al. (2014) shows the presence of a genetic cluster that can be found anywhere else on the Arabian Peninsula (Fig. 2B dark red). And (3), the sample size in our study was low (N = 3).

However, the results of previous studies on the genetic composition of *G. arabica* (Hammond et al., 2001, Wronski et al., 2010, Bärmann et al., 2013a), suggested consistent differences between eastern and western populations of Arabian gazelles. Some authors (Blacket et al., 2001, Blacket, 2001, Groves and Grubb, 2011) proposed eastern populations to represent a taxon previously described as *Antilope cora* (Smith, 1827a, Smith, 1827b), reported to originate from the coast of the Persian Gulf, i.e., eastern Arabia. The author’s imprecise original description matches rufous Arabian gazelles found in Oman and the UAE, locally known as ‘doumani’ (Fig. 1b). Unfortunately, no type specimens of original ‘*cora*’ or illustrations are available, making the designation of this taxon purely speculative and requiring a more comprehensive sampling and analysis of specimens from the UAE and Oman. Furthermore, investigations of gazelle populations in Yemen might shed some light on the question of whether those populations represent a secondary contact of two separated taxa (western and eastern populations as proposed by Wronski et al. (2010) and Bärmann et al. (2013a), or whether being the ‘missing link’ between formerly continuously distributed populations of *G. arabica*. However, the current political situation in Yemen makes such investigations improbable, if not impossible, and attempts to use museum specimens originating from that area (e.g., specimens assigned to *Gazella bilkis*; Groves and Lay, 1985) remained unsuccessful so far (Lerp et al., 2012). Nevertheless, future investigation using a larger sample size might finally shed light on the taxonomic status of eastern *G. arabica* populations.

A unique haplotype (haplotype C in Fig. 2A) was exclusively found in the wild on the Farasan Islands, suggesting a colonization of the archipelago at a time when the sea level of the Red Sea was lower, and the islands were part of the Arabian mainland (Lerp et al., 2014). This finding was further supported by highest K2P values and an exclusive cluster of population genetic data (Fig. 2B). Comparable to the colonization of gazelles on Farur Islands in the Persian Gulf (*G. a. dareshurii;* Fadakar et al., 2021), Farasan gazelles evolved separately from mainland gazelles after the sea level rose again and the island populations became isolated. While in Farur gazelles the isolation persists until today and led to a diagnosable distinct taxonomic entity (Fadakar et al., 2021), this study, as well as a previous study on Farasan gazelles (Lerp et al., 2014), suggest the introgression of mainland genes into the Farasan Island population. The Farasan gazelle population was repeatedly supplemented with gazelles from the mainland, which is more apparent in our population genetic data than in in the haplotype data. Interestingly, this result also indicates that predominantly males were introduced to the islands—otherwise other haplotypes should have been recovered, especially in those specimens with a mainland type population genetic profile (comparable to the higher mitochondrial than autosomal ancestry of cattle unravelled in bison; Hedrick, 2010). The reason for this male-biased restocking might be the horn deformations, or the complete absence of horns in female Farasan gazelles and the general smaller body sizes in Farasan gazelles compared to mainland gazelles (Lerp et al., 2014). Local people, a kind of early ship chandlers, might have selected particularly strong and well developed ‘stud males’ with well-developed horns to counteract this development and to maintain a healthy gazelle population for consumption.

### 4.3 Potential management actions

On the mainland of the Arabian Peninsula, we found no evidence for population structuring, indicating the absence of more than one conservation unit in *G. arabica*. On the Farasan Islands, however, a morphological distinguishable form of *G. arabica* exists (Lerp et al. 2014). The presence of mainland DNA in specimens carrying the unique Farasan haplotype indicates that pure ancestral Farasan gazelles cannot be detected by using the cytochrome *b* gene alone but by also including several nuclear markers into the analysis. An eligible conservation measure, i.e., the genetic restoration of the ancestral Farasan type, or restoring the population genetic structure before the population was supplemented by mainland genes, seems therefore unreasonable and impractical since all gazelles on the islands would have to be tested for cytochrome *b* and micro-satellite markers. Moreover, individuals neither carrying the Farasan haplotype C nor the unique genotype would have to be removed from the population. Such a conservation action would not just be difficult but also potentially dangerous since the population might become inviable (outbreeding depression) because it may have only survived due to the new, introduced mainland genes, preventing a higher degree of inbreeding within this isolated island population (Frankham, 1998). Furthermore, morphological skull measurements indicated a pronounced island dwarfism in all Farasan gazelles, irrespective of their genetic ancestry (Lerp et al., 2014) raising further doubts on the practicality and usefulness of such a restoration.

The captive breeding stock of *G. arabica* held at KKWRC counts about 240 individuals (80 males and 160 females) of different geographical origin within Saudi Arabia (Butynski et al., 2012). The major objective of holding such a living collection of gazelles is to maintain a viable and healthy population that provides genetically pure individuals for conservation reintroductions into the protected areas of Saudi Arabia. Our data show a good haplotype diversity (*N* = 6) within the collection, confirming the different origins of founder individuals. Apart from two haplotypes (haplotype I and J in Fig. 2A), all others present in the KKWRC breeding stock were also found in the wild, rendering the living *G. arabica* collection principally suitable for reintroductions, at least in Saudi Arabia. However, it is recommended that looking at the genetic data, i.e., the haplotypes of individuals, should facilitate future decision processes on what individuals should be introduced into what protected areas. To prevent an outbreeding depression of existing wild populations, it is further recommended to avoid releasing gazelles of mixed origin into regions where wild populations of *G. arabica* still persist. The reinforcement of existing wild populations with individuals bred in captivity should only be an option for those populations that substantially suffer from inbreeding depression (Edmands, 2007). In such cases, Edmands (2007) suggested to maximize the genetic and adaptive similarity between introduced and resident populations, and to test for the effects of hybridization for at least two generations. In Floridian panther (*Puma concolor coryi*), Johnson et al. (2010) could show that a successful genetic restauration of the Floridian population was achieved by outcrossing with Texas pumas (*Puma concolor couguar*).

For future reintroductions of Arabian gazelles in Saudi Arabia this implies, that priority should be given to regions where the species is already locally extinct. In cases where reintroduced populations are established and begin to disperse, corridors of gene flow may develop (stepping-stones), and the gene pool of the original population could be enriched, making outbreeding depressions less likely due to local adaptation of reintroduced populations. Such a viable *meta*-population was observed in Milu (*Elaphurus davidianus*), a reintroduced deer species in China, after animals dispersed into adjacent suitable habitats (Yang et al. 2016). Successful reintroductions of *Gazella arabica* using captive stock formerly held at KKWRC were already realized in three protected areas of Saudi Arabia (Uruq Bani Ma'arid: Islam et al., 2011; Mahazat as-Sayd: Sher Shah et al., 2013, Islam et al., 2014; Ibex Reserve: Dunham et al., 1993), however, with limited success since dispersing individuals leaving the protected area, often fall victim to illegal offtake by local hunters (Wronski et al., 2012). Thus, anti-hunting laws should be enforced more strictly and gazelles dispersing out of their release habitat should be monitored, e.g., by GPS collars (Dunham, 1997, Herrero et al., 2020). Only, if corridors of gene-flow between populations will be restored, the long-term survival of *G. arabica* on the Arabian Peninsula can be ensured.

## Ethical approval

The author confirm that all research activities were conducted in accordance with the relevant laws of Saudi Arabia and the institutional guidelines of KKWRC and the NCW. The authors further ensure that the appropriate institutional committee(s) have authorized the ethical standards of breeding gazelles at KKWRC and using samples for genetic analysis.

## Funding

The authors further express their gratitude to the Deputyship for Research and Innovation at the Ministry of Education in Saudi Arabia for providing the funding to realise the present research project (IFKSUOR3-535-1).

## Author contribution

The study was designed by MAM and NAH. WM and MAM collected the samples and conducted the laboratory analysis. Data analysis, and interpretation of results was done by HL, TW, and MAM. HL, TW, and MAM wrote the manuscript draft. MAB made significant revisions to the text and provided funding for the open access publication. All authors read and approved the final manuscript.

## Declaration of competing interest

The authors declare that they have no known competing financial interests or personal relationships that could have appeared to influence the work reported in this paper.
