# Supplementary material for: Revisiting the radiation of Gazella arabica on the Arabian Peninsula and testing the suitability of captive breeding stock for reintroduction, using mitochondrial and nuclear markers
Source: Saudi J Biol Sci. 2023 Oct 5;30(11):103823. doi: 10.1016/j.sjbs.2023.103823 (PMC10622698; doi:10.1016/j.sjbs.2023.103823)
Supplement: Supplementary data 1 [file mmc1.docx]

Revisiting the radiation of *Gazella arabica* on the Arabian Peninsula and testing the suitability of captive breeding stock for reintroduction, using mitochondrial and nuclear markers

**—Online Supplementary Material—**

Mohamed Al Mutairi, Hannes Lerp, Naif Al Hanosh, William Macasero and Torsten Wronski

**Table S1**: Samples used in the study with information on geographic region, origin, provenance, haplotype, Genbank Accession numbers of used gene sequences and information on available microsatellite data.

| **sample ID** | **geographic region/ species** | **origin** | **provenance** | **halpotype** | **µSats available from Lerp et al. 2014** | **Genbank Accession # Cyt b** | **Genbank Accession # NLRP2** | **Genbank Accession # PANK4** | **Genbank Accession # Smoc1** |
| --- | --- | --- | --- | --- | --- | --- | --- | --- | --- |
| 940 | north | Jordan | wild | D | x | KC188747 (Lerp et al. 2013) |  |  |  |
| 2276 | north | A'rava Valley | wild | B | x | KC188744 (Lerp et al. 2013) |  |  |  |
| 2296 | north | Al-Beda'a | wild | G | x | KC188741 (Lerp et al. 2013) |  |  |  |
| 3554 | north | A'rava Valley | wild | B | x | KC188748 (Lerp et al. 2013) |  |  |  |
| 3555 | north | A'rava Valley | wild | B | x | KC188745 (Lerp et al. 2013) |  |  |  |
| 3558 | north | A'rava Valley | wild | A | x | JN410224 (Lerp et al. 2011) |  |  |  |
| 10170 | north | A'rava Valley | wild | F | x | KC188740 (Lerp et al. 2013) | KU560749 (Lerp et al. 2016) | KU560793 (Lerp et al. 2016) | KU560883 (Lerp et al. 2016) |
| 11048 | north | A'rava Valley | wild | B | x | KC188759 (Lerp et al. 2013) | KU560750 (Lerp et al. 2016) | KU560794 (Lerp et al. 2016) | KU560884 (Lerp et al. 2016) |
| 1342KKWRC | north | Al Khunfah | wild | E |  | OR672375 (this study) | OR672219 (this study) | OR672270 (this study) | OR672323 (this study) |
| A_KSA | north | Harrat Uwarid, Saudi Arabia | wild | E | x | OR672376 (this study) |  |  |  |
| 3847 | south-west | Wadi Tarj | wild | A | x | KC188762 (Lerp et al. 2013) |  |  |  |
| 3848 | south-west | Wadi Tarj | wild | D | x | KC188766 (Lerp et al. 2013) |  |  |  |
| 351_cora | south-west | Najran, Saudi Arabia | wild | D |  | OR672377 (this study) |  |  |  |
| 466KSA | south-west | Sharurah | wild | E |  | JN410356 (Lerp et al. 2011) |  |  |  |
| Tarj1 | south-west | Wadi Tarj | wild | D | x | KC188761 (Lerp et al. 2013) |  |  |  |
| Tarj2 | south-west | Wadi Tarj | wild | A | x | OR672378 (this study) |  |  |  |
| Tarj3 | south-west | Wadi Tarj | wild | A | x | OR672379 (this study) |  |  |  |
| 2281Dubai | east | Wadi al-Safa | wild | H | x | JN410353 (Lerp et al. 2011) |  |  |  |
| OmanI | east | between Muscat and Sur | wild | H | x | KU560648 (Lerp et al. 2016) | KU560752 (Lerp et al. 2016) | KU560796 (Lerp et al. 2016) | KU560886 (Lerp et al. 2016) |
| OmanIII | east | between Muscat and Sur | wild | H | x | OR672380 (this study) |  |  |  |
| 071217FFAR | Farasan | Farasan Islands | wild | C |  | OR672381 (this study) |  | OR672271 (this study) | OR672324 (this study) |
| 071217MFAR | Farasan | Farasan Islands | wild | C |  | OR672382 (this study) |  | OR672272 (this study) | OR672325 (this study) |
| 3953_Kebir | Farasan | Farasan Kebir | wild | C | x | OR672383 (this study) |  |  |  |
| 3954_Kebir | Farasan | Farasan Kebir | wild | C | x | OR672384 (this study) |  |  |  |
| 3956_Kebir | Farasan | Farasan Kebir | wild | C | x | OR672385 (this study) |  |  |  |
| 3957_Kebir | Farasan | Farasan Kebir | wild | A | x | OR672386 (this study) |  |  |  |
| 3961_Kebir | Farasan | Farasan Kebir | wild | C | x | OR672387 (this study) |  |  |  |
| 4005FAR | Farasan | Farasan Kebir | wild | C |  | OR672388 (this study) | OR672220 (this study) | OR672273 (this study) | OR672326 (this study) |
| 584FAR | Farasan | Farasan Kebir | wild | C | x | OR672389 (this study) |  | OR672274 (this study) | OR672327 (this study) |
| 585FAR | Farasan | Farasan Kebir | wild | C | x | OR672390 (this study) | OR672221 (this study) | OR672275 (this study) | OR672328 (this study) |
| 587FAR | Farasan | Farasan Kebir | wild | C | x | OR672391 (this study) | OR672222 (this study) | OR672276 (this study) | OR672330 (this study) |

**Table S1**: continued.

| **sample ID** | **geographic region/ species** | **origin** | **provenance** | **haplotype** | **µSats available from Lerp et al. 2014** | **Genbank Accession # Cyt b** | **Genbank Accession # NLRP2** | **Genbank Accession # PANK4** | **Genbank Accession # Smoc1** |
| --- | --- | --- | --- | --- | --- | --- | --- | --- | --- |
| 588FAR | Farasan | Farasan Kebir | wild | C | x | OR672392 (this study) | OR672223 (this study) |  | OR672331 (this study) |
| 589FAR | Farasan | Farasan Kebir | wild | C | x | OR672393 (this study) | OR672224 (this study) | OR672277 (this study) | OR672332 (this study) |
| 590FAR | Farasan | Farasan Kebir | wild | C | x | OR672394 (this study) | OR672225 (this study) | OR672278 (this study) | OR672333 (this study) |
| 591FAR | Farasan | Farasan Kebir | wild | C | x | OR672395 (this study) | OR672226 (this study) | OR672279 (this study) | OR672334 (this study) |
| 714FAR | Farasan | Farasan Kebir | wild | C | x | OR672396 (this study) | OR672227 (this study) | OR672280 (this study) | OR672335 (this study) |
| 717FAR | Farasan | Farasan Kebir | wild | C | x | OR672397 (this study) | OR672228 (this study) | OR672281 (this study) | OR672336 (this study) |
| 719FAR | Farasan | Farasan Kebir | wild | C | x | OR672398 (this study) | OR672229 (this study) | OR672282 (this study) | OR672337 (this study) |
| C_Kebir | Farasan | Farasan Kebir | wild | C | x | OR672399 (this study) |  |  |  |
| D_Kebir | Farasan | Farasan Kebir | wild | C | x | OR672400 (this study) |  |  |  |
| E_Kebir | Farasan | Farasan Kebir | wild | C | x | OR672401 (this study) |  |  |  |
| F_Kebir | Farasan | Farasan Kebir | wild | C | x | OR672402 (this study) |  |  |  |
| G_Kebir | Farasan | Farasan Kebir | wild | C | x | OR672403 (this study) |  |  |  |
| GGF41 | Farasan | Farasan Kebir | wild | C | x | KU560630 (Lerp et al. 2016) | KU560751 (Lerp et al. 2016) | KU560759 (Lerp et al. 2016) | KU560885 (Lerp et al. 2016) |
| GMT8670FAR | Farasan | Farasan Islands | wild | C |  | OR672404 (this study) | OR672230 (this study) | OR672283 (this study) | OR672338 (this study) |
| GMT8671FAR | Farasan | Farasan Islands | wild | C |  | OR672405 (this study) | OR672231 (this study) | OR672284 (this study) | OR672339 (this study) |
| Jizan3447 | unknown | Jizan* | wild | C | x | JN410260 (Lerp et al. 2011) |  |  |  |
| 2383FAR | unknown | Jizan* | wild | C |  | OR672406 (this study) | OR672232 (this study) | OR672285 (this study) | OR672340 (this study) |
| 2384FAR | unknown | Jizan* | wild | C | x | OR672407 (this study) | OR672233 (this study) | OR672286 (this study) | OR672341 (this study) |
| 5 | unknown | KKWRC | captive | E | x | JN410261 (Lerp et al. 2011) |  |  |  |
| 129KKWRC | unknown | KKWRC | captive | E |  | OR672408 (this study) | OR672234 (this study) | OR672287 (this study) | OR672342 (this study) |
| 131KKWRC | unknown | KKWRC | captive | D |  | OR672409 (this study) | OR672235 (this study) | OR672288 (this study) | OR672343 (this study) |
| 132KKWRC | unknown | KKWRC | captive | E |  | OR672410 (this study) |  | OR672289 (this study) | OR672344 (this study) |
| 135KKWRC | unknown | KKWRC | captive | A |  | OR672411 (this study) |  | OR672290 (this study) | OR672345 (this study) |
| 136KKWRC | unknown | KKWRC | captive | D |  | OR672412 (this study) | OR672236 (this study) | OR672291 (this study) | OR672346 (this study) |
| 137KKWRC | unknown | KKWRC | captve |  |  |  | OR672237 (this study) | OR672292 (this study) | OR672347 (this study) |
| 146KKWRC | unknown | KKWRC | captive | A |  | OR672413 (this study) | OR672238 (this study) | OR672293 (this study) | OR672348 (this study) |
| 1533KKWRC | unknown | KKWRC | captive | E |  | OR672414 (this study) | OR672239 (this study) | OR672294 (this study) | OR672349 (this study) |
| 1622KKWRC | unknown | KKWRC | captive | E |  | OR672415 (this study) | OR672240 (this study) | OR672295 (this study) |  |
| 165KKWRC | unknown | KKWRC | captive |  |  |  | OR672241 (this study) | OR672296 (this study) | OR672350 (this study) |
| 170KKWRC | unknown | KKWRC | captive | A |  | OR672416 (this study) | OR672242 (this study) |  | OR672351 (this study) |
| 173KKWRC | unknown | KKWRC | captive | E |  | OR672417 (this study) | OR672243 (this study) | OR672297 (this study) | OR672352 (this study) |
| 177KKWRC | unknown | KKWRC | captive | A |  | OR672418 (this study) | OR672244 (this study) | OR672298 (this study) | OR672353 (this study) |
| 1802KKWRC | unknown | KKWRC | captive | I |  | OR672419 (this study) | OR672245 (this study) | OR672299 (this study) | OR672354 (this study) |
| 180KKWRC | unknown | KKWRC | captive | E |  | OR672420 (this study) | OR672246 (this study) | OR672300 (this study) | OR672355 (this study) |
| 1950KKWRC | unknown | KKWRC | captive | C |  | OR672421 (this study) | OR672247 (this study) | OR672301 (this study) |  |
| 1957KKWRC | unknown | KKWRC | captive | J |  | OR672422 (this study) | OR672248 (this study) | OR672302 (this study) | OR672356 (this study) |
| 1984KKWRC | unknown | KKWRC | captive | E |  | OR672423 (this study) | OR672249 (this study) | OR672303 (this study) | OR672357 (this study) |
| 2056KKWRC | unknown | KKWRC | captive | E |  | OR672424 (this study) | OR672250 (this study) | OR672304 (this study) | OR672358 (this study) |
| 2080KKWRC | unknown | KKWRC | captive | E |  | OR672425 (this study) | OR672251 (this study) | OR672305 (this study) | OR672359 (this study) |
| 2104KKWRC | unknown | KKWRC | captive | I |  | OR672426 (this study) | OR672252 (this study) | OR672306 (this study) | OR672360 (this study) |
| 2151KKWRC | unknown | KKWRC | captive | E |  | OR672427 (this study) | OR672253 (this study) | OR672307 (this study) | OR672361 (this study) |
| 2187KKWRC | unknown | KKWRC | captive | D |  | OR672428 (this study) | OR672254 (this study) | OR672308 (this study) | OR672362 (this study) |

**Table S1**: continued.

| **sample ID** | **geographic region/ species** | **origin** | **provenance** | **haplotype** | **µSats available from Lerp et al. 2014** | **Genbank Accession # Cyt b** | **Genbank Accession # NLRP2** | **Genbank Accession # PANK4** | **Genbank Accession # Smoc1** |
| --- | --- | --- | --- | --- | --- | --- | --- | --- | --- |
| 2261KKWRC | unknown | KKWRC | captive | C |  | OR672429 (this study) | OR672255 (this study) | OR672309 (this study) | OR672363 (this study) |
| 2262KKWRC | unknown | KKWRC | captive | E |  | OR672430 (this study) | OR672256 (this study) | OR672310 (this study) | OR672364 (this study) |
| 2344KKWRC | unknown | KKWRC | captive | A |  | OR672431 (this study) | OR672257 (this study) | OR672311 (this study) | OR672365 (this study) |
| 3296KKWRC | unknown | KKWRC | captive | A |  | OR672432 (this study) | OR672258 (this study) | OR672312 (this study) | OR672366 (this study) |
| 3297KKWRC | unknown | KKWRC | captive | A |  | OR672433 (this study) | OR672259 (this study) | OR672313 (this study) |  |
| 3298KKWRC | unknown | KKWRC | captive | A |  | OR672434 (this study) |  |  |  |
| 3299KKWRC | unknown | KKWRC | captive | A |  | OR672435 (this study) | OR672260 (this study) | OR672314 (this study) | OR672367 (this study) |
| 3300KKWRC | unknown | KKWRC | captive | A |  | OR672436 (this study) | OR672261 (this study) | OR672315 (this study) | OR672368 (this study) |
| 3301KKWRC | unknown | KKWRC | captive | E |  | OR672437 (this study) | OR672262 (this study) | OR672316 (this study) |  |
| 3304KKWRC | unknown | KKWRC | captive | A |  | OR672438 (this study) | OR672263 (this study) |  |  |
| 3436KKWRC | unknown | KKWRC | captive | E |  | OR672439 (this study) | OR672264 (this study) | OR672317 (this study) | OR672369 (this study) |
| 3437KKWRC | unknown | KKWRC | captive | D |  | OR672440 (this study) | OR672265 (this study) | OR672318 (this study) | OR672370 (this study) |
| 3439KKWRC | unknown | KKWRC | captive |  |  |  | OR672266 (this study) | OR672319 (this study) | OR672371 (this study) |
| 3441KKWRC | unknown | KKWRC | captive | E |  | OR672441 (this study) | OR672267 (this study) | OR672320 (this study) | OR672372 (this study) |
| 637KKWRC | unknown | KKWRC | captive | A |  | OR672442 (this study) | OR672268 (this study) | OR672321 (this study) | OR672373 (this study) |
| 662KKWRC | unknown | KKWRC | captive | E |  | OR672443 (this study) | OR672269 (this study) | OR672322 (this study) | OR672374 (this study) |
| 8_yemeni | unknown | KKWRC | captive | E | x | JN410356 (Lerp et al. 2011) |  |  |  |
| Katit1 | unknown | KKWRC | captive | C |  | OR672444 (this study) |  |  |  |
| GH1 | *G. gazella* |  | wild |  |  | KU560629 (Lerp et al. 2016) | KU560746 (Lerp et al. 2016) | KU560790 (Lerp et al. 2016) | KU560880 (Lerp et al. 2016) |
| TAUM11861 | *G. gazella* |  | wild |  |  | KC188775 (Lerp et al. 2013) | KU560747 (Lerp et al. 2016) | KU560791 (Lerp et al. 2016) | KU560881 (Lerp et al. 2016) |
| TAUM12479 | *G. gazella* |  | wild |  |  | KC188774 (Lerp et al. 2013) | KU560748 (Lerp et al. 2016) | KU560792 (Lerp et al. 2016) | KU560882 (Lerp et al. 2016) |
| 2866 | *G. dorcas* |  | wild |  |  | JN410252 (Lerp et al. 2011) | KU560759 (Lerp et al. 2016) | KU560805 (Lerp et al. 2016) | KU560891 (Lerp et al. 2016) |
| Chad19 | *G. dorcas* |  | wild |  |  | JN410237 (Lerp et al. 2011) | KU560758 (Lerp et al. 2016) | KU560803 (Lerp et al. 2016) | KU560889 (Lerp et al. 2016) |

**References**

Lerp, H., Wronski, T., Pfenninger, M. & Plath, M. (2011) A phylogeographic framework for the conservation of Saharan and Arabian dorcas gazelles. Organisms Diversity and Evolution 11(4): 317-329.

Lerp, H., Torsten Wronski, Martin Plath, Anne Schröter, Markus Pfenninger (2013) Phylogenetic and population genetic analyses suggest a potential species boundary between Mountain (*Gazella gazella*) and Arabian Gazelles (*G. arabica*) in the Levant, Mammalian Biology, Volume 78, Issue 5, Pages 383-386.

Lerp H, Plath M, Wronski T, Malczyk A, Riesch RR, Streit B, Pfenninger M (2014) Utility of island populations in reintroduction programs—relationships between Arabian gazelles (*Gazella arabica*) from the Farasan Archipelago and endangered mainland populations. Molecular Ecology 23: 1910-1922.

Lerp, H, Klaus, S., Allgöwer, S., Wronski, T., Pfenninger, M. and Plath, M (2016) Phylogenetic analysis of true gazelles reveals repeated transitions of key ecological traits and provides novel insights into the origin of the genus *Gazella*. Molecular Phylogenetics and Evolution. 98: 1–10, doi: 10.1016/j.ympev.2016.01.012
